# Label-free quantitative proteomic profiling reveals differential plasma protein expression in patients with obesity after treatment with liraglutide

**DOI:** 10.3389/fmolb.2024.1458675

**Published:** 2024-09-11

**Authors:** Afshan Masood, Hicham Benabdelkamel, Salini Scaria Joy, Abdulaziz Alhossan, Bashayr Alsuwayni, Ghalia Abdeen, Madhawi Aldhwayan, Nora A. Alfadda, Alexander Dimitri Miras, Assim A. Alfadda

**Affiliations:** ^1^ Proteomics Resource Unit, Obesity Research Center, College of Medicine, King Saud University, Riyadh, Saudi Arabia; ^2^ Strategic Center for Diabetes Research, College of Medicine, King Saud University, Riyadh, Saudi Arabia; ^3^ Corporate of Pharmacy Services, King Saud University Medical City, Riyadh, Saudi Arabia; ^4^ Department of Clinical Pharmacy, College of Pharmacy, King Saud University, Riyadh, Saudi Arabia; ^5^ Department of Community Health Sciences, Clinical Nutrition, College of Applied Medical Sciences, King Saud University, Riyadh, Saudi Arabia; ^6^ Section of Investigative Medicine, Division of Diabetes, Endocrinology and Metabolic Medicine, Hammersmith Hospital, Imperial College London, London, United Kingdom; ^7^ School of Medicine, Ulster University, Derry, United Kingdom; ^8^ Department of Medicine, College of Medicine, King Saud University, Riyadh, Saudi Arabia

**Keywords:** obesity, liraglutide, GLP1 receptor agonist, proteomics, label-free quantification, multimerin-2, BPI fold-containing family a member 2

## Abstract

**Introduction:**

Treatment and management of obesity is clinically challenging. The inclusion of GLP-1 receptor agonists (GLP1RA) in the medical management of obesity has proven to be efficacious. However, mechanisms underlying the molecular changes arising from GLP1RA treatment in patients with obesity remain to be elucidated.

**Methods:**

A single-center, prospective study was undertaken to evaluate the changes in the plasma proteins after liraglutide 3 mg therapy in twenty patients (M/F: 7/13) with obesity (mean BMI 40.65 ± 3.7 kg/m^2^). Anthropometric and laboratory parameters were measured, and blood samples were collected at two time points: baseline, before initiating treatment (pretreatment group, PT), and after three months of receiving the full dose liraglutide 3 mg (posttreatment group, PoT). An untargeted label-free LC MSMS mass spectrometric approach combined with bioinformatics and network pathway analysis was used to determine changes in the proteomic profiles.

**Results:**

The mean age of the study participants was 36.0 ± 11.1 years. A statistically significant change was observed in weight, BMI and HbA1c levels between the PT and PoT groups (paired t-test, *P* < 0.001). A significant dysregulation was noted in the abundances of 151 proteins (31 up and 120 downregulated) between the two groups. The potential biomarkers were evaluated using receiver operating characteristic (ROC) curves. The top ten proteins (area under the curve (AUC) of 0.999 (95% CI)) were identified as potential biomarkers between PT and PoT groups and included Cystatin-B, major vault protein, and plastin-3, which were upregulated, whereas multimerin-2, large ribosomal P2, and proline–rich acidic protein 1 were downregulated in the PoT group compared with the PT group. The top network pathway identified using ingenuity pathway analysis (IPA), centered around dysregulation of MAPK, AKT, and PKc signaling pathways and related to cell-to-cell signaling and interaction, cellular assembly and organization, cellular compromise and a score of 46 with 25 focus proteins.

**Discussion:**

Through label-free quantitative proteomic analysis, our study revealed significant dysregulation of plasma proteins after liraglutide 3 mg treatment in patients with obesity. The alterations in the proteomic profile between the PT and PoT groups demonstrated a decrease in levels of proteins involved in inflammation and oxidative stress pathways. On the other hand proteins involved in the glycolytic and lipolytic metabolic pathways as well as those participating in cytoskeletal and endothelial reorganization were observed to be increased. Understanding actions of liraglutide at a molecular and proteomic levels provides a holistic look into how liraglutide impacts metabolism, induces weight loss and improves overall metabolic health.

## 1 Introduction

Treatment and management of obesity has been a challenge worldwide, and overcoming obesity is among the top priorities of many countries. The initial paradigm that obesity was simply a condition that can be resolved successfully by creating a state of negative energy balance through lifestyle intervention and diet was largely unsuccessful in sustaining long-term weight loss. The focus of obesity management does not solely rely on weight reduction but also on improving metabolic outcomes by decreasing the risk of developing associated comorbid conditions, such as diabetes, hypertension, dyslipidemia, metabolic dysfunction-associated steatotic liver disease (MASLD), sleep disorders, cardiovascular disease (CVD), and cancer. These comorbidities detrimentally impact health outcomes, potentially leading to decreased lifespan or accelerated aging (Michos et al., 2023).

As with any chronic disease, pharmacotherapy plays a crucial role in the management of obesity, as treatments have an effective and scalable approach. In the last decade, medical management of obesity has been at the forefront with increased use of incretin-based therapy, which includes GLP1RA therapy. Randomized clinical trials (RCTs), including the SCALE, LEAD and LEAN trials, have established the role of GLP1RAs on weight loss and weight management with and without associated comorbidities (Madsbad, 2009; Nauck et al., 2009; Pi-Sunyer et al., 2015; Armstrong et al., 2016). Liraglutide, a long-acting GLP1RA that shares 97% similarity with native mature GLP-1, has demonstrated remarkable efficacy in randomized clinical trials, with 63.2% of individuals achieving clinically significant weight loss of at least 5% of baseline body weight at 1 year (Davies et al., 2015; Pi-Sunyer et al., 2015). A sustained loss of weight for up to 2 years was also noted in patients who remained on continuous treatment with liraglutide, although discontinuing treatment could lead to weight gain (Astrup et al., 2012; Wadden et al., 2013).

The pharmacological effects of liraglutide are multifaceted and, like the native GLP-1, exert their action after binding to G protein-coupled receptors (GPCRs) in target cells or tissues. Binding of the GLP-1 to its receptor effectively coordinates signals from multiple organs and cells through the cAMP signaling cascade. These actions result in a multitude of functions, resulting in improved glucose regulation, enhanced insulin secretion, decreased glucagon secretion, controlled food intake and increased satiety, thereby delaying gastric emptying (Imeryuz et al., 1997; Muscogiuri et al., 2014; Alruwaili et al., 2021) and increasing energy expenditure (Osaka et al., 2005; Lockie et al., 2012). In addition to these benefits, previous studies have shown that liraglutide has a cardioprotective effect, directly or indirectly as observed in the levels of risk factors (blood pressure, cholesterol levels, postprandial triglyceride and glucose levels, coagulability, and inflammation) and improvements in overall metabolic control (Hsieh et al., 2010; Wajcberg and Amarah, 2010; Mikhail, 2014).

Proteomics combined with mass spectrometry provides a comprehensive overview of changes in protein abundance and dynamics occurring systemically and across various disease conditions and states. The insights gained from these alterations in turn help to understand the underlying molecular mechanisms based on protein-protein interactions and the dysregulation of signaling pathways. Proteomics also enables the identification of protein biomarkers associated with obesity-related complications, aiding in risk stratification and treatment monitoring. This approach characterizes different proteins based on their levels of expression, functions and interactions. Studies using high throughput mass spectrometry enable us to examine the differences in proteome from different states, including the pretreatment and post-treatment states. Given the complexity of the action of liraglutide, which involves multiple pathways and organs, an untargeted proteomic approach is better suited for investigating its molecular mechanisms (Astrup et al., 2012). We could identify only a limited number of studies that characterized the changes in the proteomic profiles of patients with obesity after treatment with liraglutide. Our group, in our previously published studies, demonstrated changes in the plasma and urinary proteomes of patients with diabetes after treatment with liraglutide 1.8 mg, (Ekhzaimy et al., 2022; Rafiullah et al., 2023).

In this study, we employed the LC-MSMS label free quantitative bottoms up approach with data dependent analysis to determine the protein expression profiles and metabolic changes occurring in patients before and after receiving treatment with liraglutide. The alterations in the proteomics profile was then explored using bioinformatics analysis to identify the highly connected proteins and their associated central nodes that are modulated with treatment and/or weight loss. Understanding these changes at cellular levels that effect overall metabolism with liraglutide treatment need to be ascertained.

## 2 Materials and methods

### 2.1 Ethical considerations and informed consent

The study protocol and procedures used in the study were approved by the Institutional Review Board, College of Medicine, King Saud University (no. E- 21-5853), prior to undertaking the study. All the participants provided written informed consent. The study was performed in accordance with the ethical standards of the Declaration of Helsinki and the universal International Conference on Harmonization-Good Clinical Practice Guidelines.

### 2.2 Study subjects and sample collection

We recruited twenty patients with obesity who were followed by their primary physician in the obesity clinic at King Saud University Medical City. All patients were class 2 obese (BMI >35 kg/m^2^), had no associated comorbidities, were drug naïve and had no contraindications to the use of liraglutide. The treatment was prescribed by their physician as a scaled-up dosing regimen from 0.6 mg to 3.0 mg liraglutide over a period of 5 weeks, and the drug was administered once daily via subcutaneous injection. They were followed up prospectively until they completed 12 weeks of treatment with liraglutide 3 mg. Blood samples were collected from the patients after a 10 h fast before initiating therapy (pretreatment, PT) and after completing 12 weeks of treatment (posttreatment, PoT). The plasma was separated by centrifugation (15 min, 3,000 × g), divided into several aliquots, and stored at −80°C for further analysis. Laboratory analyses of fasting blood glucose, lipid levels, liver function and CRP levels were carried out at both time points.

### 2.3 Biochemical analyses

Biochemical analyses were carried out via a Dimension Xpand Plus integrated clinical chemistry autoanalyzer (Siemens Healthcare Diagnostics, IL, United States). HbA1c was analysed using high-performance liquid chromatography and ion-exchange chromatography (normal range 4.3%–5.8%; Tosoh Bioscience, San Francisco, United States). The laboratory data are presented as the means ± SDs. Statistical significance of the difference between two groups was analysed by paired Student’s t test, with a value of *p* < 0.05 considered as statistically significant.

### 2.4 Sample preparation for proteomics

#### 2.4.1 Depletion of abundant proteins

Stored plasma samples were taken and thawed at 25°C–30°C. The samples were vortexed for 1–3 min and centrifuged for 2–3 min at 2,000 × g. For the experiment, 15 µL of plasma sample was taken, and 0.6 μL of protease inhibitor (25X) was added to each sample. Following this, high abundance plasma proteins (albumin, immunoglobulins, alpha-1 antitrypsin, and transferrin) that might interfere with MS analysis were depleted using Pierce™ Top 12 Abundant Protein Depletion Spin Columns (Thermo Fisher Scientific, Waltham, MA, United States) according to the manufacturer’s instructions and protocol. Proteins were extracted via 4x ice-cold acetone precipitation. The protein concentration of each sample was determined in triplicate using 2D-Quant Kit (GE Healthcare, Piscataway, NJ, United States).

#### 2.4.2 Protein digestion and peptide quantification

Fifty micrograms of plasma proteins were transferred to a tube containing 10 µL of urea denaturing buffer (6 M urea). Disulfide bonds from the plasma proteins were reduced by adding 1 µL of dithiothreitol (200 mM), and the mixture was incubated for 30 min at 60°C. Afterward, the samples were alkylated by adding 1 µL of iodoacetamide (400 mM) solution and incubated at room temperature for another 30 min in the dark. The samples were diluted with 65 µL of ammonium bicarbonate buffer (50 mM) and digested overnight at 37 °C by adding 2.5 µL of sequencing-grade modified trypsin (Promega, United States) (1 μg/μL). To acidify the samples, 7 µL of 10% formic acid was added, and subsequently, the samples were desalted using Pierce C18 spin columns (Thermo Scientific, United States), and the peptide concentration was determined using a Pierce Quantitative Colorimetric Peptide Assay (Thermo Scientific™ Pierce) (Müller et al., 2012; Kumar et al., 2020). The peptides eluted from the Pierce C18 spin were dried via vacuum centrifugation (Eppendorf Concentrator plus TM, Eppendorf, Germany).

### 2.5 Liquid chromatography coupled with tandem mass spectrometry (LC‒MS/MS)

Peptides were reconstituted in a solution containing 0.1% (v/v) formic acid, and then, 1 µL of each sample was applied to a Dionex UltiMate 3,000 nano LC system with a WPS-3000 autosampler. The peptide was injected and concentrated on a PepMap100 C18 trapping column (3 μm, 100 Å, 75 µm 285 inner diameter [i.d.] × 20 mm, nanoViper; Thermo Scientific) that was equilibrated with 0.05% TFA 286 in water. The selective trapping of nano-LC achieved high-capacity sample loading. After the trap column was switched inline, LC separations were performed via an analytical column (PepMap™ C18, 50 cm × 75 μm). Peptide separation was carried out at 300 nL/min with mobile phase A, which consisted of 0.1% (v/v) formic acid in water, while mobile phase B contained 0.1% (v/v) formic acid and 80% (v/v) acetonitrile in water. The column was preequilibrated with 5% mobile phase B, followed by an increase to 22.5% mobile phase B over 139 min and then 45% mobile phase B over 184 min. After separation, the peptides were injected into the nanospray ion source for ionization and then analyzed by a Q Exactive Plus Hybrid Quadrupole-Orbitrap mass spectrometer (Thermo Fisher Scientific, United States) operating in positive ion mode, with a nanoelectrospray (nESI) potential of 2,000 V and a maximum duty cycle of 3s. The scanning range for primary mass spectrometry (MS) was set to 375–1,650 m/z, and the scanning resolution was set to 70,000; the fixed starting point of the scanning range for secondary mass spectrometry (MS/MS) was set to 80 m/z, and the scanning resolution for MS/MS spectrometry was set to 17,500. The dynamic exclusion time was set to 20 s, and the automatic gain control was set to 3 × 10^6^ and 1 × 10^5^ for the MS and MS/MS scans, respectively. Mass spectra were acquired in data-dependent acquisition mode (DDA).

### 2.6 Data processing

MS and MS/MS raw data analysis was performed using Proteome Discoverer v3.0 (Thermo Fisher Scientific, Bremen, Germany) with Sequest as the search engine and HUMAN-refprot-isoforms.fasta as the sequence database. The parameters applied for the analysis were as follows: 1) precursor/fragment mass tolerance: 15 ppm/0.02 Da; 2) maximal missed cleavages: 2; 3) enzyme name: trypsin (Full); 4) dynamic modifications: peptide N-terminal acetylation and methionine oxidation; and 5) static modification: cysteine carbamidomethylation. Peptide-spectrum match (PSM) filtering was conducted with the protein and peptide FDRs set at 1%, with a minimum of two unique peptides per protein. The filtered PSM list was exported and manually formatted in Excel. Multivariate statistical analysis was performed using MetaboAnalyst v. 6.0 (McGill University, Montreal, QC, Canada) (http://www.metaboanalyst.ca, accessed on 12 March 2024) (Benabdelkamel et al., 2024). Only proteins that were identified and quantified with LFQ intensity were used for downstream analysis.

### 2.7 Statistical and bioinformatics analysis

All statistical comparisons between groups were performed using student’s t-test implemented by Proteome Discoverer v3.0. Adjusted *P*-values after FDR (q-values) were considered significant for values less than 0.01. The differently expressed significant proteins (FDR *p*-value ≤0.05, and a fold change ≥1.5) were exported from the Proteome Discoverer. Ingenuity pathway analysis (IPA) was carried out by importing quantitative data into the Qiagen IPA software (QIAGEN Inc., https://digitalinsights.qiagen.com/IPA). This software aids in determining the functions and pathways that are most strongly associated with the protein list by overlaying the experimental expression data on networks constructed from published interactions. Furthermore, the PANTHER (protein analysis through evolutionary relationships) classification system (http://www.pantherdb.org) was used to categorize the identified proteins based on their molecular function and biological process.

## 3 Results

### 3.1 Clinical and biochemical data

The baseline characteristics of the study group are presented in Table 1. The mean age of the study participants was 36 ± 11.1 years. After treatment with liraglutide 3 mg, we observed significant changes in body weight, BMI, and HbA1c levels (paired t-test, *p*-value < 0.05). We did not observe any significant changes in the other metrics.

**TABLE 1:** Clinical and biochemical characteristics of the study population before and after treatment with liraglutide 3 mg.

	Pretreatment (n = 20)	Posttreatment with liraglutide 3 mg (n = 20)	*p*-value
Age (years)	36.0 ± 11.1 years		
Weight (kg)	115.07 ± 13.48	106.6 ± 11.92	**<0.001**
BMI (kg/m^2^)	41.96 ± 4.91	39.07 ± 5.29	**<0.001**
ALT (IU/L)	33.15 ± 16.75	32.65 ± 12.41	0.860
AST (IU/L)	20.15 ± 8.2	17.40 ± 5.91	0.133
HbA1c (%)	5.74 ± 0.43	5.35 ± 0.35	**<0.001**
Cholesterol (mmol/L)	4.84 ± 1.2	4.76 ± 0.35	0.650
HDL (mmol/L)	1.34 ± 0.34	1.33 ± 0.4	0.847
LDL (mmol/L)	2.76 ± 0.93	2.66 ± 0.92	0.560
Triglycerides (mmol/L)	0.98 ± 0.6	1.18 ± 0.9	0.234
Insulin (mIU/L)	19.8 ± 14.1	19.13 ± 9.8	0.254
CRP (mg/L)	8.57 ± 8.4	7.41 ± 6.4	0.136

The values are expressed as the means ± standard deviations (paired t tests, *P* values < 0.05). BMI: body mass index, Hb: hemoglobin, WBC: white blood cell, RBC: red blood cell, PLT: platelet, ESR: erythrocyte sedimentation rate, ALT: alanine aminotransferase, AST: aspartate transaminase, HDL: high-density lipoprotein, LDL: low-density lipoprotein, HbA1C: glycated hemoglobin, CRP: C-reactive protein. The bold values indicate *p* values significant at level less than 0.05.

### 3.2 Proteomic analysis and identification of differentially expressed proteins

#### 3.2.1 Label-free quantitative proteomic analysis

Label-free quantitative proteomics was used to compare samples from the two groups (PoT and PT). In total, 1,019 nonredundant proteins were quantified by identifying one or more unique peptides. Significant and differentially expressed proteins were defined as those that presented a fold change greater than 1.5 or less than 0.66 in relative abundance and a *P*-value <0.05. On the basis of these criteria, we identified 151 proteins (120 up- and 31 downregulated) as significantly differentially expressed between the PoT and PT groups.

#### 3.2.2 Analysis of differentially expressed proteins in response to liraglutide treatment

**FIGURE 1:**
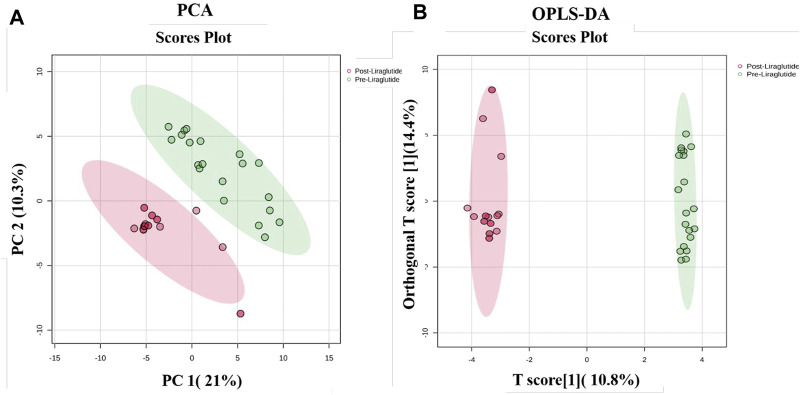
Multivariate analysis of proteomic profiles of patients with obesity between the PoT and PT groups. **(A)** Two-dimensional principal component analysis (PCA) score plot and **(B)** OPLS-DA score plot showing a clear separation between the two groups, indicating significant proteomic differences between them. The robustness of the created models was evaluated by the fitness of the model (R2Y = 0.97) and predictive ability (Q2 = 0.898) values in a larger dataset (n = 100).

The proteins that distinguished between the PT and PoT groups are displayed in Figure 1. The visualization of each study group and outlier detection were carried out via principal component analysis (PCA). Figure 1A. The PCA model demonstrated a clear separation between the proteomic profiles of the PT and PoT groups. The samples were colored according to the color of their group. Additionally, orthogonal partial least squares discriminant analysis (OPLS-DA), a supervised multivariate approach, was applied to the dataset and revealed a distinct separation between the protein profiles of (PoT and PT) (Figure 1B). The robustness of the created models was evaluated by the fitness of the model (R2Y = 0.97) and predictive ability (Q2 = 0.898) values in a larger dataset (n = 100).

**FIGURE 2:**
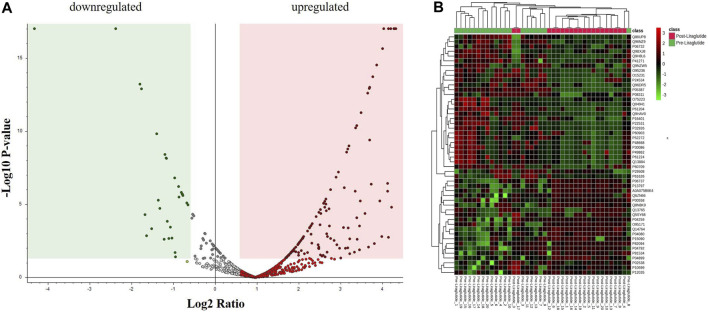
**(A)** Volcano plot showing a significant change in the levels of several proteins, of which red represents upregulated and green represents downregulated plasma proteins in the posttreatment and preliraglutide treatment groups (FDR *p*-value ≤0.05-fold change ≥1.5). **(B)** Hierarchical clustering (HAC) and heatmap analysis of identified proteins that were significantly altered between the post- and preliraglutide treatment groups. The color range bar indicates downregulated proteins as green and upregulated proteins as red.

A moderate t-test (*p*-value <0.05) and fold change (FC cutoff of 1.5) were used to analyze the volcano plot between the PoT and PT treatment groups. Among the 151 dysregulated proteins, 120 (red) and 31 (green) proteins were upregulated and downregulated between the post- and preliraglutide treatment groups (Figure 2A; Supplementary Data S1). The intensity changes of the 151 differentially expressed proteins are shown as a heatmap in Figure 2B. Thus, these proteins might be considered possible protein biomarkers for identifying the molecular, biological and cellular changes that occur after liraglutide treatment.

#### 3.2.3 Evaluation of protein biomarkers between study groups

**FIGURE 3:**
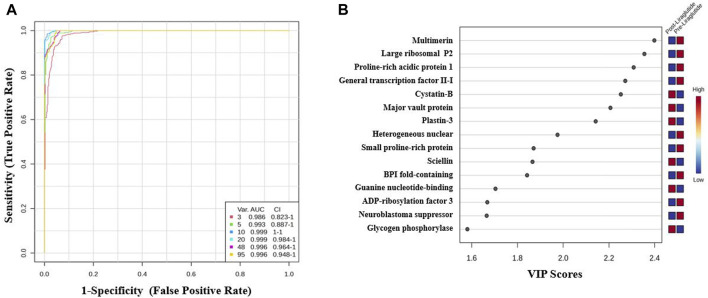
Biomarker evaluation of the significantly dysregulated proteins between the PoT and PT groups. **(A)** Receiver operating characteristic (ROC) curve generated by the OPLS-DA model, with area under the curve (AUC) values calculated from the combination of 3, 5, 10, 20, 48, and 95 proteins. **(B)** Frequency plot showing the 15 significantly dysregulated identified protein biomarkers in the post- and preliraglutide treatment groups.

The potential biomarkers were evaluated via receiver operating characteristic (ROC) curves. PLS-DA was used as a classification and feature ranking approach to create a multivariate exploratory ROC analysis. Ten features at the exploratory ROC curve using PLS-DA with cross-validation (CV) had an area under the curve (AUC) value of at least 0.999 (95% CI) (Figure 3A). A frequency plot of the top 15 significantly dysregulated protein biomarkers in the PoT and PT groups revealed that Cystatin-B, Major vault protein, and plastin-3 were upregulated in the PoT group, whereas, Multimerin, Large ribosomal P2, Proline–rich acidic protein 1 were downregulated in the PoT group compared with the PT group (Figure 3B).

#### 3.2.4 Evaluation of protein biomarkers in the postliraglutide and preliraglutide treatment groups

**FIGURE 4:**
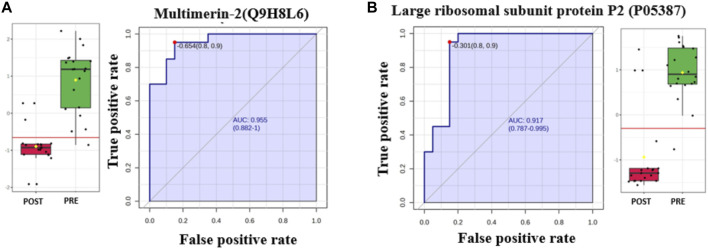
Two downregulated proteins in the postliraglutide treatment group with the highest AUC: **(A)** Multimerin-2 (Q9H8L6), AUC = 0.955; box plot (FDR *p* ≤ 0.05 and fold change ≥1.5), where red represents postliraglutide treatment and green represents preliraglutide treatment; **(B)** Large ribosomal subunit protein P2 (P05387), AUC = 0.917; box plot (FDR *p* ≤ 0.05 and fold change ≥1.5), where red represents the postliraglutide treatment group and green represents the preliraglutide treatment group.

**FIGURE 5:**
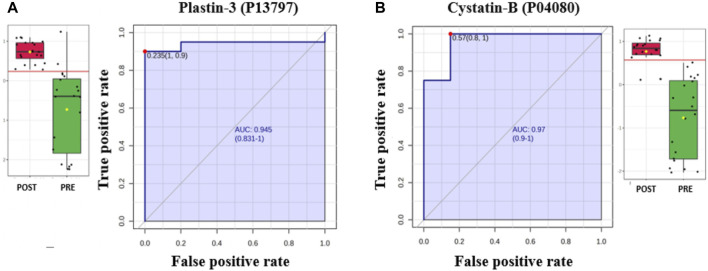
Two proteins upregulated in the postliraglutide treatment group with the highest AUC: **(A)** Plastin-3 (P13797), AUC = 0.945; box plot (FDR *p* ≤ 0.05 and fold change ≥1.5), where red represents the postliraglutide treatment group and green represents the preliraglutide treatment group. **(B)** Cystatin-B (P04080), AUC = 0.97; box plot (FDR *p* ≤ 0.05 and fold change ≥1.5), where red represents the postliraglutide group and green represents the preliraglutide treatment group.

The AUC of the ROC curve for Multimerin-2 (Q9H8L6) was 0.955 (Figure 4A), that for large ribosomal subunit protein P2 (P05387) (Figure 4B) was 0.917, and both were downregulated after liraglutide treatment. The box and whisker plots revealed decreased expression of Multimerin and large ribosomal subunit protein P2 in the postliraglutide treatment group compared with the preliraglutide treatment group. The area under the ROC curve (AUC) values for Plastin -3 (P13797) (Figure 5A) and Cystatin -B (P04080) (Figure 5B) were 0.945 and 0.97, respectively, after liraglutide treatment. The box and whisker plots revealed increased expression of Plastin-3 and Cystatin-B in the postliraglutide treatment group compared with the preliraglutide treatment group.

#### 3.2.5 Interaction network analysis of differentially expressed proteins

**FIGURE 6:**
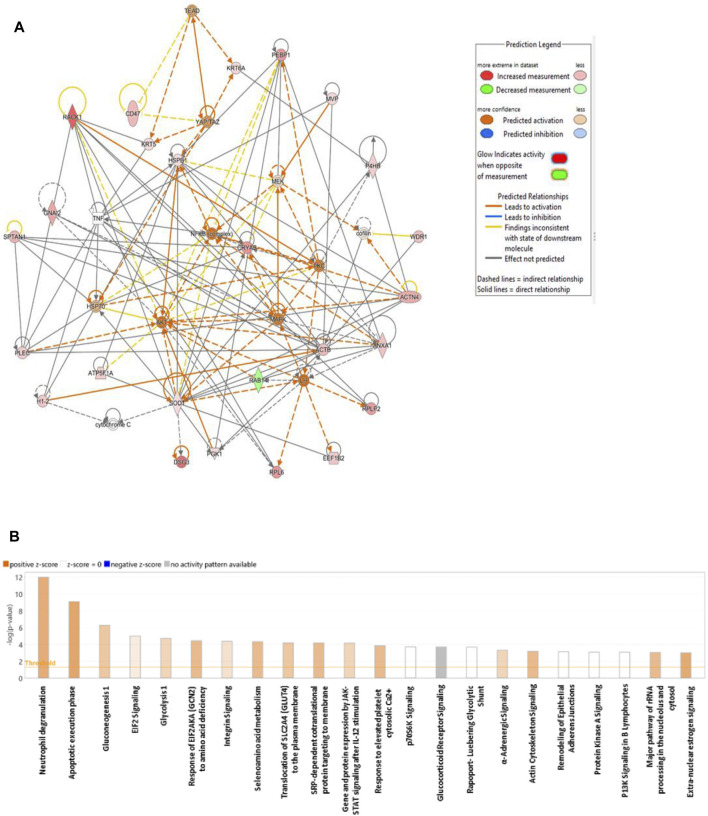
Schematic representation of the highest scoring network pathways depicting the involvement of the differentially regulated proteins. Nodes colored green represent downregulation, and those colored red represent upregulation. **(A)** Protein interaction network pathway between the post- and preliraglutide treatment groups. **(B)** Top canonical pathways ranked by the *p* values obtained by IPA. The blue bars represent negative z scores, the orange bars represent positive z scores, and the gray bars indicate that no activity pattern was available. The interaction networks were generated through IPA (QIAGEN, Inc.).

The biological impact of the changes in abundances of proteins in our data set were examined using IPA software. The software computes a score based on the best fit obtained from the input dataset of proteins and from the biological functions database, to generate a protein–protein interactions network. The protein network with protein interactions are shown in Figure 6A. The significant proteins in our data set were related to 10 network pathways. Among these two overlapping networks showed the highest score. The top network pathway centered around dysregulation of MAPK, AKT, and PKc, signaling pathways (Figure 6A) and related to cell-to-cell signaling and interaction, cellular assembly and organization, cellular compromise with a score of 46 with 25 focus proteins. The second pathway demonstrated a score of 41 with 23 proteins and centered around dysregulation of NFkb, ERK1/2, MAP2K ½, pI3K, and p38K signalling pathways and related to cell death and survival, cell-to-cell signalling and interaction, cellular function and maintenance. The canonical pathways related to the altered proteins in the data set related to neutrophil degranulation, apoptotic execution phase, gluconeogenesis, EIF2 signaling, glycolysis, etc., were the most significant pathway with a positive z score (Figure 6B) (Supplementary Data S2).

**FIGURE 7:**
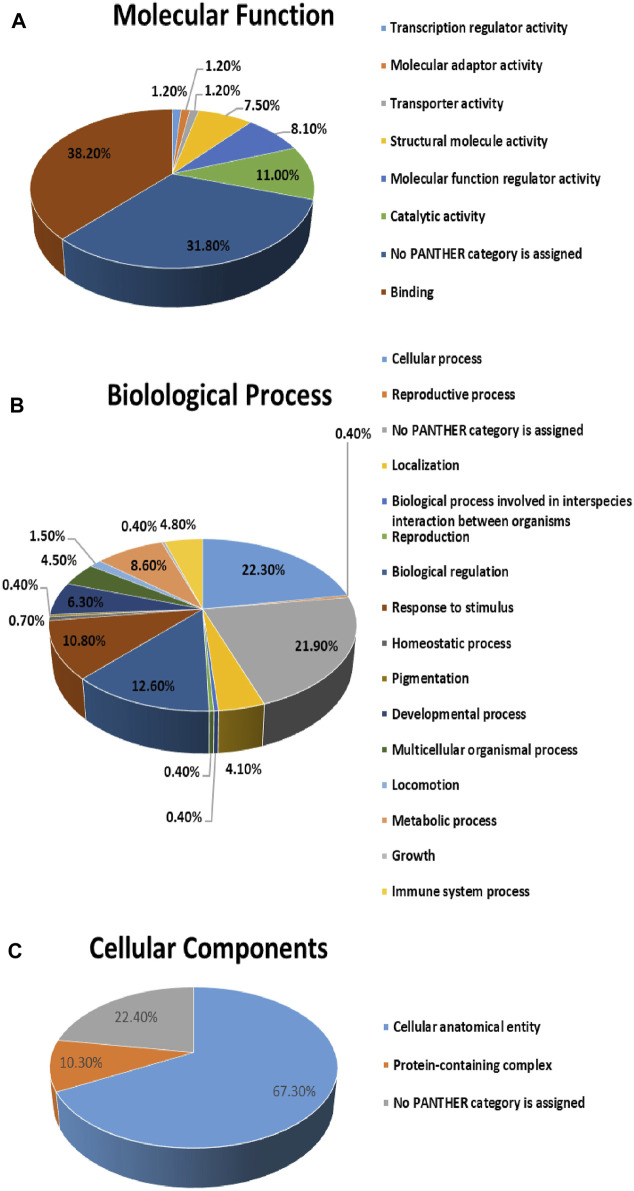
Comparative depiction of identified proteins categorized into groups according to their molecular functions **(A)**, biological processes **(B)** and **(C)** cellular components.

The protein analysis through evolutionary relationships (PANTHER) classification system was used to identify the proteins by their molecular functions (Figure 7A), biological processes (Figure 7B) and cellular components (Figure 7C). The functional category showed that most of the differentially expressed proteins identified were enzymes with binding (38.2%) followed by catalytic activity (11.0%) (Figure 7A). With regards to biological processes, the identified proteins were involved in cellular process (22.3%) and biological regulation (12.6%) (Figure 7B). The majority of the identified proteins were located in the cellular anatomical entity (67.3%), followed by the protein containing complex (10.3%) (Figure 7C).

## 4 Discussion

In the present study an untargeted label free quantitative proteomic profiling was conducted to identify changes in protein pattern between patients before and after receiving liraglutide 3 mg for 12 weeks. In general, the PoT group showed significant and clinically meaningful decrease in weight, BMI, HbA1c levels. The weight loss in our study is comparable with other large, randomized, controlled study that showed reduction in body weight by 8.4 ± 7.3 kg in adult patients (Pi-Sunyer et al., 2015). Although liraglutide has been documented as an effective tool in the treatment and management of obesity it’s use has shown additional metabolic benefits in reducing cardiovascular risk factors and fatty liver disease (Dhir et al., 2003; Honigberg et al., 2020). Our results showed a clear and distinct separation in the abundance of 151 (31 decreased and 120 increased) significantly differentially regulated proteins between the two groups. GO enrichment and bioinformatic analyses of the significantly differentially regulated proteins identified that majority of them participated in regulation of inflammatory response (including neutrophil degranulation), carbohydrate metabolism (including glycolysis, gluconeogenesis, glycogenolysis) and lipid metabolism, oxidative stress and in cytoskeletal reorganization.

### 4.1 Dysregulation of proteins involved in the inflammatory response

Our study identified significant dysregulation of proteins involved in the regulation of the inflammatory response and the functions of different immune cells in the PT and PoT groups. It is well known that inflammatory responses determine metabolic pathophysiological outcomes in the diseased microenvironment. Low-grade systemic inflammation has been recognized as the underlying cause of the development of chronic diseases, including obesity, diabetes, and cardiovascular disease, and also contributes to the progressive worsening of patient conditions. Additionally, inflammatory processes play important roles in the pathophysiology of obesity, insulin resistance (IR) and the modulation of insulin signaling (Gao et al., 2015).

These strong interactions between the immune system and metabolism are central to immunometabolism, which is influenced by liraglutide. Although the induction of immune cells is known to increase inflammation and thereby increase IR, alternatively activated immune cells, including neutrophils and macrophages, attenuate tissue inflammation and reduce IR in obese mice (Odegaard and Chawla, 2008; Vauclard et al., 2023). Here, we identified proteins that are involved in the regulation of neutrophils and macrophages in the two groups. The top dysregulated canonical pathway identified by IPA analysis between the PT and PoT groups was related to neutrophil degranulation. Among the 21 proteins involved in this process, 6 proteins (FTL, CKAP4, RAB14, RAB7A, RAP1B, and SERPINB3) were downregulated, and 15 proteins (CRISP3, S100A7, LYZ, MVP, ANXA2, DSG1, PGAM1, AZU1, CD47, CTSG, SPTAN1, CSTB, ACTR2, GPI, and PYGL) were upregulated. Neutrophils play a major role in regulating inflammation through the release of neutrophil extracellular trap networks (NETs) via NETosis (Herrero-Cervera et al., 2022; Zhang and Liu, 2024), a recently discovered anti-inflammatory mechanism (Schauer et al., 2014). This process results in the release of many granular and anti-inflammatory proteins, including azurocidin, whose levels were increased in the PoT group. The increase in the neutrophil degranulation pathway related to the identified proteins in our dataset indicates that liraglutide may regulate neutrophils, similar to that observed in animal models (Toki et al., 2021), and potentially enhance their inflammatory actions.

In addition to neutrophils, macrophages are well known to promote a low-grade chronic inflammatory state in obesity and associated vascular dysfunction. An increase in the levels of major vault protein (MVP), expressed by macrophages endothelial cells and hepatocytes, was noted in the PoT group. MVP is the main component of cellular ribonucleoprotein particles that are implicated in the regulating, signaling transduction, cellular differentiation, cell survival, and immune responses and metabolic inflammation (Berger et al., 2009; Ben et al., 2019). MVP deficiency, in animal models was associated with increased plasma levels of nonesterified fatty acids, triglycerides, and total cholesterol, and also aggravated obesity and associated IR, hepatic steatosis and atherosclerosis in mice (Ben et al., 2019). The increase in the levels of neutrophil degranulation and MVP with liraglutide treatment could be one of the many ways GLP1RA demonstrate potent anti-inflammatory potentials from their impact on immune cell signaling. Additional in-depth investigation into the underlying molecular pathways is required.

### 4.2 Dysregulation of proteins involved in carbohydrate and lipid metabolism

We detected significant differences in the levels of enzymes involved in regulating glucose homeostasis and lipid metabolism between the two groups. The levels of GPI, PGAM1, PGK1, and MDH2 were increased, whereas those of BPGM were decreased in the PoT group. The identified proteins are involved in regulating glycolytic and gluconeogenic metabolic pathways and the tricarboxylic acid cycle (TCA). The increase in proteins regulating the glycolytic pathways and the TCA cycle with liraglutide treatment indicates increased cellular metabolic activity and decreased glucose storage. Interestingly, in addition to its role in the TCA cycle, MDH2 acts as a mitochondrial metabolic checkpoint in the TCA cycle, and oxidative phosphorylation drives these metabolic pathways (Molinie et al., 2022). MDH2 has also been shown to play a critical role in insulin secretion, and mutations in this gene are known to cause diabetes (Jungtrakoon Thamtarana et al., 2022). It has been suggested that GLP-1 induces insulin secretion through several molecular pathways and that the upregulation of these proteins, including MDH2, could be one of these mechanisms. We also detected a decrease in the levels of the enzyme lactoyl glutathione lyase, also known as glyoxalase1, a key enzyme in the glyoxalase pathway in the PoT group. Downregulation of GLO1 in an animal model was associated with reduced adiposity and ectopic fat accumulation, which is in line with the reduced weight observed in our patients after liraglutide treatment (Silhavy et al., 2020). Our results also revealed an increase in the abundance of glycogen phosphorylase, an enzyme that catalyzes the first step of glycogenolysis to release glucose-1-phosphate monomers from glycogen, further promoting glucose breakdown and impeding glucose storage.

In addition to carbohydrate metabolism, proteins related to lipid metabolism were also significantly dysregulated between the PT and PoT groups. An increase in the levels of FABP4 and ApoL3 and a decrease in the level of PRAP1, which are proteins that bind to fatty acids and mobilize them across different cell membranes, were detected. Fatty acid binding protein 4 (FABP4), or adipocyte P2 (aP2), is an adipokine produced by adipocytes and macrophages that critically integrates metabolic and inflammatory responses from both types of cells to regulate obesity. FABP4 impacts both lipolysis and insulin secretion through the adipo-pancreatic axis (Scheja et al., 1999). It binds intracellular fatty acids and mediates intracellular lipid trafficking between cellular compartments. Knockdown of FABP4 was shown to result in reduced lipid transport efficiency. FABP4 also has insulinotropic potential, as it stimulates β cells and alters insulin secretion to maintain glucose homeostasis (Parkes et al., 2001; Wu et al., 2014). Another significant protein identified in our study was BPI, which contains a fold protein. BPI is a lipid transfer protein secreted from adipocytes and the liver, with increased levels in obese individuals and additional anti-inflammatory actions (Sun et al., 2010; Moreno-Navarrete et al., 2012).

### 4.3 Dysregulation of proteins involved in regulating oxidative stress

An increase in the levels of antioxidants superoxide dismutase, thioredoxin, and protein disulfide-isomerase were noted in the PoT group compared to PT. Oxidative stress is a well-known feature of obesity that is a consequence of increased white adipose tissue and increased circulating free fatty acids. Superoxide dismutase (SOD) is a major endogenous cytoprotective antioxidant enzymes present in humans that defends against free oxygen radicals. It prevents endothelial and mitochondrial dysfunction by inactivating nitric oxide and inhibiting peroxynitrite formation (Fukai and Ushio-Fukai, 2011). As an antioxidant metalloenzyme, SOD catalyzes the disproportionation of superoxide anion radicals to generate oxygen and hydrogen peroxide, which plays an essential role in the fight against oxidative stress.

Besides the oxidative stress, obesity along with chronic inflammation involving inflammatory cell infiltration can trigger endoplasmic reticulum (ER) stress leading to IR (Tripathi and Pandey, 2012; Ajoolabady et al., 2022). Along with SOD we identified an increase in levels of two proteins belonging to the thioredoxin superfamily of redox proteins namely thioredoxin (TRX), and protein disulfide isomerase (PDI). Redox regulation by TRX and PDI plays a crucial role in biological responses against oxidative stress (Tanaka et al., 2000; Dunn et al., 2021), specifically in the endoplasmic reticulum stress response, and is crucial for the antioxidant balance of endothelial cells, among other functions (Wan et al., 2012). Proteolipid protein 2 (PLP2), an integral ion channel membrane protein of the endoplasmic reticulum, plays an important role in this process. In animal models, *PLP2* knockout increased ER stress and induced apoptotic cell death (Breitwieser et al., 1997; Zhang et al., 2015; Feng et al., 2020). We noted an increase in PLP2 levels after treatment, indicating that liraglutide ameliorates ER stress. The antioxidant capacity is well known to be reduced in patients with obesity, and an increase in the levels of these enzymes in the PoT group indicates that liraglutide treatment ameliorates oxidative stress. Our findings are in line with other studies that have shown that GLP-1 treatment improves insulin sensitivity by attenuating oxidative stress (Gao et al., 2015) in both animal models and in clinical studies (Vincent and Taylor, 2006; Okada et al., 2014; Rizzo et al., 2015; Song et al., 2022). This evidence strongly suggests that GLP-1 improves oxidative stress and thereby improves clinical outcomes, in terms of reducing hepatic inflammation and improving insulin sensitivity.

### 4.4 Dysregulation of proteins involved in cytoskeletal reorganization and endothelial dysfunction

A significant increase was noted among the proteins involved in the regulation of cytoskeletal structure and the extracellular matrix. Cytoskeletal reorganization is important for modulating cell shape and size; for the formation of cellular projections (e.g., desmosomes), cellular junctions and secretory vesicles; and for cell adhesion. Obesity alters the cytoskeletal architecture of immune cells and adipocytes by influencing their size and altering their secretory profile. Remodeling of the cytoskeletal membrane and extracellular matrix (ECM) composition is necessary for adipose tissue expansion and cell migration. Both increase adipocyte size (hypertrophy) and inappropriate extracellular matrix remodeling (fibrosis) contributes to the pathogenesis of adipose tissue dysfunction in individuals with obesity (Crewe et al., 2017). Many proteins involved in this process were identified in our study. These include actin, Myosin regulatory light chain 12B, annexins, Desmoglein (DSM1, 3), Integrin alpha-IIb, keratins (prelamin A/C, PDI, Plakophilin-3, plastin-3 and plectin). Actin and its related proteins are the main components of the cytoskeleton, whose dynamic formation critically influences physiological cellular processes, such as focal adhesion, cell motility, endo- and exocytosis, cell polarity, signal transduction, and intracellular trafficking, mechanotransduction and cell division (Dhir et al., 2003). Annexins are Ca^2+^-dependent phospholipid-binding proteins, that are also involved in signal transduction, cell motility, cell adhesion and reducing inflammatory response (Shao et al., 2019; Xi et al., 2020). Another feature of this remodelling is an epithelial/endothelial-mesenchymal transition (EMT). Epithelial cells line the surfaces of all organs and tissues, including within adipose tissue, while endothelial cells line the vasculature. EMT is a complex biological process whereby epithelial/endothelial cells acquire the invasive, migratory mesenchymal phenotype (Chavkin et al., 2023). Two cytoskeletal proteins, namely sciellin, plectin involved in this transitioning were identified in our study. These cytoskeletal proteins are capable of interlinking different elements of the cytoskeleton,and like other cytoskeletal proeins play crucial roles in maintaining cell and tissue integrity and orchestrating dynamic changes in cytoarchitecture and cell shape, and serve as scaffolding platforms for the assembly, positioning, and regulation of signaling complexes (Chou et al., 2016; Song et al., 2022). A previous study showed that Sciellin increases the mesenchymal to epithelial transitions and promotes epithelial phenotype. Reducing cancer cell migration and invasion abilities in patients with colorectal cancer and hepatic metastasis (Chou et al., 2016). On the other hand plectin downregulation was found to decrease migration and EMT (Xu et al., 2022). More mechanistic studies are need to understand how GLP1RA including liraglutide affect this transitioning. An increase in the level of multimerin-2, an extracellular matrix glycoprotein predominantly found in endothelial cells that is a key molecule required to maintain vascular homeostasis stability in vasculogenesis, angiogenesis and cell matrix adhesion, was noted. It is known to regulate the VEGFA/VEGFR2 signaling axis and angiogenesis in response to cellular hypoxia and inflammation to modulate the vascularity and tissue remodeling of adipose tissue. Depletion of multimerin-2 leads to increased EC permeability (Pellicani et al., 2020).

### 4.5 Network analysis of biological pathways related to liraglutide treatment and biomarker analysis

To gain insights into the biological impact associated with our set of differentially regulated proteins a network pathway analysis was performed using IPA. This approach identified the protein–protein interactions and provided additional insights into the most common pathways associated with our set of proteins. Network pathway analysis identified that the significantly dysregulated proteins centered around dysregulation of mitogen activated protein kinase (MAPK) family (MAPK, MEK, PKc, AKT) and SOD signaling pathways which were noted to be activated in the activity map. PKC-MAPK pathways are involved in several cellular responses, and are important for mounting appropriate responses to stress, regulation of appetite, and glucose homeostasis. Previous studies showed that regulation of MAPK/PKc/AKT pathways is impotant in regulation of glucose, membrane translocation of glucose transporters and insulin signaling (Chen et al., 2021; Zhu et al., 2023). Inhibition of AKT expression or signaling leads to impaired insulin-stimulated glucose transport and resulted in IR in both muscle and liver (Li et al., 2022; Wen et al., 2022). Liraglutide treatment was also noted to increase activity of SOD pathway that is an important mechanism in combating oxidative stress.

A simultaneous biomarker analysis of our data set was carried out using ROC curves to characterize proteins with a higher specificity and sensitivity to liraglutide treatment. Multivariate ROC analysis showed that a panel of Ten proteins (Large ribosomal subunit protein P2, Multimerin-2, BPI fold-containing family A member 2, Plastin -3, Cystatin B, general transcription factor II-I repeat domain-containing protein 2A, ADP-ribosylation factor 3, sciellin, GP, PRAP) contributed to the highest AUC of 0.999. The identification of these proteins is significant as they collectively indicate changes in the modulation of extracellular matrix, and immune cell movement especially neutrophils (sciellin, multimerin-2, plastin-3, and cystatin B), lipid metabolism (GP, PRAP, and BPI) and nucleic acid metabolism (Large ribosomal subunit protein P2, general transcription factor II-I repeat domain-containing protein 2A, ADP-ribosylation factor 3) post treatment with liraglutide 3 mg.

## 5 Conclusion

In conclusion, our present study using label free quantitative proteomics revealed significant differences in the plasma proteomic profiles of patients with obesity before and after treatment with liraglutide 3 mg. Treatment with liraglutide resulted in significant weight loss, decrease in abundance of proteins associated with inflammation and oxidative stress, and an increase in proteins involved in glycolytic, and lipolytic metabolism, cytoskeletal reorganization and endothelial remodeling pathways. The multitude of actions across different tissues can be explained by the ubiquitous nature of the GLP1 receptors to which it binds. Alterations in levels of the identified proteins could help to get a deeper understanding of effects of liraglutide treatment on metabolic pathways that induce weight loss and improve metabolic health.

## Data Availability

The datasets presented in this study can be found in online repositories. The names of the repository/repositories and accession number(s) can be found below: MassIVE repository via accession ID: MSV000095740.

## Supplementary material

SUPPLEMENTARY DATA S1
Details of the significantly differentially expressed proteins between the PT and PoT groups.

SUPPLEMENTARY DATA S2
The biological impact of the changes in the expression of proteins in our dataset was examined via IPA software.
