# Supplementary material for: Label-free quantitative proteomic profiling reveals differential plasma protein expression in patients with obesity after treatment with liraglutide
Source: Front Mol Biosci. 2024 Sep 11;11:1458675. doi: 10.3389/fmolb.2024.1458675 (PMC11422103; doi:10.3389/fmolb.2024.1458675)
Supplement: Supplementary file 1 [file DataSheet2.pdf]

Analysis Name: Saxenda IPA_2.1 - 2024-04-08 11:02 pm
Analysis Creation Date: 2024-04-08
Build version: exported
Content version: 111725566 (Release Date: 2024-03-21)

Experiment Metadata

Name	Value

Analysis Settings

Reference set: Ingenuity Knowledge Base (Genes Only)
Relationship to include: Direct and Indirect
Does not Include Endogenous Chemicals
Optional Analyses: My Pathways My List

Filter Summary:
Consider only molecules and/or relationships where
(species = Rat OR Human OR Mouse) AND
(tissues = Adipose OR Other Granulocytes OR Esophagus OR Effector memory cytotoxic T cells OR Salivary Gland OR Immature monocyte-derived dendritic cells OR B lymphocytes not otherwise specified OR Adipocytes OR Brain OR Bone marrow-derived dendritic cells OR Other NK cells OR Activated CD56bright NK cells OR Endothelial cells not otherwise specified OR Effector memory helper T cells OR Peripheral blood monocytes OR Thalamus OR Other Monocytes OR Activated CD56dim NK cells OR Activated helper T cells OR Activated Vd1 Gamma-delta T

(c) 2000-2024 QIAGEN. All rights reserved.

cells OR Other Bone marrow cells OR Spleen OR Blood platelets OR Prostate Gland OR Stomach OR Small Intestine OR Hippocampus OR Other Nervous System OR Peripheral blood lymphocytes OR Cardiomyocytes OR Monocytes not otherwise specified OR Other Tissues and Primary Cells OR Testis OR Gray Matter OR Pre-B lymphocytes OR Peritoneal macrophages OR CD56dim NK cells OR Dendritic cells not otherwise specified OR Mast cells OR Other Immune cells OR Other B lymphocytes OR Lymphocytes not otherwise specified OR Thymus OR Kidney OR Ovary OR Granule Cell Layer OR Peripheral blood leukocytes not otherwise specified OR Th17 cells OR Lung OR Central memory helper T cells OR Plasmacytoid dendritic cells OR Other Lymphocytes OR Hypothalamus OR Other Mononuclear leukocytes OR Immune cells not otherwise specified OR Naive B cells OR Other Smooth muscle cells OR Retina OR Murine NKT cells OR Th2 cells OR Uterus OR Memory T lymphocytes not otherwise specified OR CD56bright NK cells OR Microvascular endothelial cells OR Trachea OR Activated Vd2 Gamma-delta T cells OR Effector T cells OR Mature monocyte-derived dendritic cells OR Beta islet cells OR Other Macrophages OR Pro-B lymphocytes OR Putamen OR Cartilage Tissue OR CD4+ T-lymphocytes OR Crypt OR Smooth Muscle OR Vd1 Gamma-delta T cells OR Heart OR Bone marrow cells not otherwise specified OR Subventricular Zone OR Thyroid Gland OR Skin OR Osteoblasts OR Myeloid dendritic cells OR Other Monocyte-derived dendritic cells OR Substantia Nigra OR Nucleus Accumbens OR Medulla Oblongata OR Choroid Plexus OR Effector memory RA+ cytotoxic T cells OR NK cells not otherwise specified OR Cytotoxic T cells OR Thymocytes OR Other Endothelial cells OR Memory B cells OR Brainstem OR Granulocytes not otherwise specified OR Other T lymphocytes OR Placenta OR Olfactory Bulb OR Other Peripheral blood leukocytes OR PBMCs OR Trigeminal Ganglion OR Cerebral Ventricles OR BDCA-1+ dendritic cells OR Stem cells not otherwise specified OR Dermis OR Pituitary Gland OR Large Intestine OR Eosinophils OR Vascular smooth muscle cells OR Cornea OR Parietal Lobe OR Lymph node OR CD34+ cells OR Bone marrow-derived macrophages OR Naive helper T cells OR Calvaria OR Caudate Nucleus OR Pancreas OR Other Stem cells OR Splenocytes OR Microglia OR T lymphocytes not otherwise specified OR Epidermis OR Embryonic stem cells OR Natural T-regulatory cells OR Cerebellum OR Monocyte-derived dendritic cells not otherwise specified OR Megakaryocytes OR Astrocytes OR HUVEC cells OR Plasma cells OR Stromal cells OR Mononuclear leukocytes not otherwise specified OR Forestomach OR Ventricular Zone OR Central memory cytotoxic T cells OR Chondrocytes OR Vd2 Gamma-delta T cells OR Other Organ Systems OR Neutrophils OR Th1 cells OR Langerhans cells OR Mammary Gland OR Nervous System not otherwise specified OR Cerebral Cortex OR Other Cells OR Striatum OR Corpus Callosum OR Fibroblasts OR Adrenal Gland OR Tissues and Primary Cells not otherwise specified OR BDCA-3+ dendritic cells OR Liver OR Organ Systems not otherwise specified OR Other Memory T lymphocytes OR White Matter OR Macrophages not otherwise specified OR Dorsal Root Ganglion OR Sciatic Nerve OR Bladder OR Smooth muscle cells not otherwise specified OR Other Dendritic cells OR Spinal Cord OR Cells not otherwise specified OR Lens OR Mesenchymal stem cells OR Intraepithelial T lymphocytes OR Amygdala OR Skeletal Muscle OR Hematopoietic progenitor cells OR Monocyte-derived macrophage OR Granulosa cells) AND

(mol. types = biologic drug OR canonical pathway OR chemical - kinase inhibitor OR chemical - protease inhibitor OR complex OR cytokine OR disease OR enzyme OR function OR fusion gene/product OR G-protein coupled receptor OR group OR growth factor OR ion channel OR kinase

(c) 2000-2024 QIAGEN. All rights reserved.

INGENUITY PATHWAY ANALYSIS

OR ligand-dependent nuclear receptor OR other OR peptidase OR phosphatase OR transcription regulator OR translation regulator OR transmembrane receptor OR transporter) AND
(data sources = An Open Access Database of Genome-wide Association Results OR BIND OR BioGRID OR Catalogue Of Somatic Mutations In Cancer (COSMIC) OR Chemical Carcinogenesis Research Information System (CCRIS) OR Clinical Genome Resource (ClinGen) OR ClinicalTrials.gov OR ClinVar OR Cognia OR DIP OR DrugBank OR Gene Ontology (GO) OR GVK Biosciences OR Hazardous Substances Data Bank (HSDB) OR HumanCyc OR Ingenuity Expert Findings OR Ingenuity ExpertAssist Findings OR IntAct OR Interactome studies OR MIPS OR miRBase OR miRecords OR Mouse Genome Database (MGD) OR Obesity Gene Map Database OR Online Mendelian Inheritance in Man (OMIM) OR Reactome OR TarBase OR TargetScan Human OR TargetScan Mouse)

Top Canonical Pathways

Name	p-value	Overlap
**Keratinization**	1.37E-14	7.9 % 17/214
**Neutrophil degranulation**	1.02E-12	4.4 % 21/476
**Apoptotic execution phase**	8.13E-10	15.4 % 8/52
**Gluconeogenesis I**	5.19E-07	18.5 % 5/27
**Eukaryotic Translation Elongation**	1.69E-06	7.4 % 7/95

Top Upstream Regulators

Upstream Regulators

Name	p-value	Predicted Activation
**MYC**	2.20E-08	Activated

(c) 2000-2024 QIAGEN. All rights reserved.

**MAPT**	4.11E-08
**KRT14**	4.52E-08
**MLXIPL**	1.11E-07
**CAV1**	3.30E-06

Causal Network

Name	p-value	Predicted Activation
**TSC2**	1.48E-14	
**JAK3**	6.82E-14	
**INSR**	1.04E-11	
**GZMB**	2.07E-11	
**DLG1**	2.54E-11	

Top Diseases and Bio Functions

Diseases and Disorders

Name	p-value range	# Molecules
**Organismal Injury and Abnormalities**	1.78E-02 - 7.81E-12	139
**Dermatological Diseases and Conditions**	1.78E-02 - 2.41E-11	108
**Immunological Disease**	1.72E-02 - 2.48E-08	82
**Inflammatory Disease**	1.34E-02 - 4.66E-08	58
**Respiratory Disease**	1.78E-02 - 2.16E-07	89

(c) 2000-2024 QIAGEN. All rights reserved.

INGENUITY PATHWAY ANALYSIS

Molecular and Cellular Functions

Name	p-value range	# Molecules
Cell Death and Survival	1.78E-02 - 7.81E-12	60
Cellular Movement	1.78E-02 - 8.01E-11	59
Cellular Function and Maintenance	1.78E-02 - 2.45E-06	59
Cell-To-Cell Signaling and Interaction	1.78E-02 - 3.36E-06	38
Protein Synthesis	9.00E-03 - 6.48E-06	42

Physiological System Development and Function

Name	p-value range	# Molecules
Hematological System Development and Function	1.78E-02 - 1.14E-07	41
Immune Cell Trafficking	1.78E-02 - 1.14E-07	26
Skeletal and Muscular System Development and Function	1.78E-02 - 7.21E-06	20
Organismal Survival	3.46E-03 - 1.39E-05	54
Cardiovascular System Development and Function	1.78E-02 - 9.58E-05	27

Top Tox Functions

Assays: Clinical Chemistry and Hematology

(c) 2000-2024 QIAGEN. All rights reserved.

INGENUITY
PATHWAY ANALYSIS

Name	p-value range	# Molecules
**Increased Levels of Red Blood Cells**	3.47E-02 - 3.47E-02	3
**Increased Levels of Bilirubin**	8.06E-02 - 8.06E-02	1
**Increased Levels of Potassium**	1.02E-01 - 1.02E-01	1
**Increased Levels of Hematocrit**	1.23E-01 - 1.23E-01	2
**Increased Levels of Alkaline Phosphatase**	3.39E-01 - 3.39E-01	1

Cardiotoxicity

Name	p-value range	# Molecules
**Cardiac Dysfunction**	2.98E-01 - 6.54E-07	16
**Cardiac Arrythmia**	4.17E-01 - 2.58E-04	8
**Tachycardia**	4.11E-02 - 2.58E-04	3
**Cardiac Enlargement**	2.28E-01 - 3.71E-03	10
**Cardiac Necrosis/Cell Death**	1.39E-01 - 3.85E-03	6

Hepatotoxicity

Name	p-value range	# Molecules
**Hepatocellular carcinoma**	2.73E-01 - 9.02E-05	29
**Liver Hyperplasia/Hyperproliferation**	1.00E00 - 9.02E-05	63
**Liver Inflammation/Hepatitis**	4.04E-01 - 5.98E-03	4
**Liver Steatosis**	2.55E-01 - 5.98E-03	8
**Liver Enlargement**	2.59E-02 - 2.59E-02	3

(c) 2000-2024 QIAGEN. All rights reserved.

Nephrotoxicity

Name	p-value range	# Molecules
**Renal Damage**	4.90E-01 - 2.43E-04	6
**Renal Tubule Injury**	2.43E-04 - 2.43E-04	5
**Glomerular Injury**	2.59E-01 - 1.19E-02	4
**Renal Inflammation**	4.67E-01 - 2.37E-02	3
**Renal Nephritis**	4.67E-01 - 2.37E-02	3

Top Regulator Effect Networks

ID	Regulators	Disease & Functions	Consistency Score
**1**	BHLHE40,TSC2	Chemotaxis,Inflammation of organ,Necrosis of tumor (+1 more)	3.536
**2**	MYC	Cell death of tumor cells	-6.0
**3**	BHLHE40	Inflammation of body cavity	-7.5
**4**	RNASEH2B	Inflammation of body cavity	-8.083

Top Networks

ID	Associated Network Functions	Score
**1**	Cell-To-Cell Signaling and Interaction, Cellular Assembly and Organization, Cellular Compromise	46

(c) 2000-2024 QIAGEN. All rights reserved.

**2**	Cellular Function and Maintenance, Cell-To-Cell Signaling and Interaction, Cell Death and Survival	41
**3**	Neurological Disease, Organismal Injury and Abnormalities, Nervous System Development and Function	27
**4**	Cell Death and Survival, Organismal Injury and Abnormalities, Cellular Movement	27
**5**	Dermatological Diseases and Conditions, Organismal Injury and Abnormalities, Cellular Movement	25

Top Tox Lists

Name	p-value	Overlap
**Recovery from Ischemic Acute Renal Failure (Rat)**	3.08E-03	14.3 % 2/14
**Acute Renal Failure Panel (Rat)**	6.13E-03	4.8 % 3/62
**Persistent Renal Ischemia-Reperfusion Injury (Mouse)**	1.39E-02	6.7 % 2/30
**NRF2-mediated Oxidative Stress Response**	1.44E-02	2.1 % 5/238
**Cardiac Necrosis/Cell Death**	1.99E-02	1.7 % 6/354

(c) 2000-2024 QIAGEN. All rights reserved.

INGENUITY PATHWAY ANALYSIS

Top My Lists

Top My Pathways

Name	p-value	Overlap
**Network 2**	1.15E-15	16.7 % 13/78

Top ML Disease Pathways

Name	p-value	Overlap
**Blood clot**	1.30E-03	8.3 % 3/36
**Early onset cardiomyopathy**	1.30E-03	8.3 % 3/36
**Acute pancreatitis**	1.76E-03	7.5 % 3/40
**Development of hemangioma**	1.89E-03	7.3 % 3/41
**Ptosis**	1.89E-03	7.3 % 3/41

Top Analysis-Ready Molecules

Expr Fold Change

(c) 2000-2024 QIAGEN. All rights reserved.

Molecules	Expr. Value	Chart
MDH2	↑ 18.653	
BPIFA1	↑ 16.138	
NACA	↑ 15.916	
SUMO2	↑ 15.385	
SPRR2E	↑ 14.561	
RACK1	↑ 14.024	
IDE	↑ 13.303	
DSG3	↑ 12.981	
PYGL	↑ 12.978	
HMGB2	↑ 12.909	

Expr Fold Change

Molecules	Expr. Value	Chart
GGCT	↓ -5.181	
GTF2IRD2/GTF2IRD2B	↓ -3.472	
FTL	↓ -3.390	
CKAP4	↓ -3.195	
ITGA2B	↓ -3.125	
LEP	↓ -2.865	
RAB14	↓ -2.625	
PALS2	↓ -2.564	
GLO1	↓ -2.500	
IGHV3-64D	↓ -2.320	

(c) 2000-2024 QIAGEN. All rights reserved.

INGENUITY PATHWAY ANALYSIS
